# A realistic evaluation: the case of protocol-based care

**DOI:** 10.1186/1748-5908-5-38

**Published:** 2010-05-26

**Authors:** Jo Rycroft-Malone, Marina Fontenla, Debra Bick, Kate Seers

**Affiliations:** 1 Centre for Health Related Research, School of Healthcare Sciences, Bangor University, Ffriddoedd Road, Bangor, UK; 2 RCN Research Institute, School of Health and Social Studies, University of Warwick, Coventry, UK; 3 Florence Nightingale School of Nursing and Midwifery, King's College London, James Clerk Maxwell Building, 57 Waterloo Road, London, UK

## Abstract

**Background:**

'Protocol based care' was envisioned by policy makers as a mechanism for delivering on the service improvement agenda in England. Realistic evaluation is an increasingly popular approach, but few published examples exist, particularly in implementation research. To fill this gap, within this paper we describe the application of a realistic evaluation approach to the study of protocol-based care, whilst sharing findings of relevance about standardising care through the use of protocols, guidelines, and pathways.

**Methods:**

Situated between positivism and relativism, realistic evaluation is concerned with the identification of underlying causal mechanisms, how they work, and under what conditions. Fundamentally it focuses attention on finding out what works, for whom, how, and in what circumstances.

**Results:**

In this research, we were interested in understanding the relationships between the type and nature of particular approaches to protocol-based care (mechanisms), within different clinical settings (context), and what impacts this resulted in (outcomes). An evidence review using the principles of realist synthesis resulted in a number of propositions, *i.e.*, context, mechanism, and outcome threads (CMOs). These propositions were then 'tested' through multiple case studies, using multiple methods including non-participant observation, interviews, and document analysis through an iterative analysis process. The initial propositions (conjectured CMOs) only partially corresponded to the findings that emerged during analysis. From the iterative analysis process of scrutinising mechanisms, context, and outcomes we were able to draw out some theoretically generalisable features about what works, for whom, how, and what circumstances in relation to the use of standardised care approaches (refined CMOs).

**Conclusions:**

As one of the first studies to apply realistic evaluation in implementation research, it was a good fit, particularly given the growing emphasis on understanding how context influences evidence-based practice. The strengths and limitations of the approach are considered, including how to operationalise it and some of the challenges. This approach provided a useful interpretive framework with which to make sense of the multiple factors that were simultaneously at play and being observed through various data sources, and for developing explanatory theory about using standardised care approaches in practice.

## Background

This paper explores the application of realistic evaluation as a methodological framework for an evaluation of protocol-based care. The United Kingdom's National Health Service (NHS) has been on its modernisation journey for over 10 years [1], during which time there has been considerable investment in an infrastructure to support a vision of high quality service provision [2]. The promotion of 'protocol-based care' was envisaged as one mechanism for delivering on the modernisation agenda (through standardisation of practice) and for strengthening the co-ordination of services across professional and environmental boundaries (through role blurring) [2,3]. It was anticipated by the Department of Health that by 2004 the majority of staff would be working under agreed protocols [2].

However, whilst there has been sustained political enthusiasm for protocol-based care, no systematic evaluation of its impact had been undertaken; particularly across multiple care sectors and services. Subsequently, the National Institute for Health Research's Service Delivery and Organisation Programme funded research into how protocol-based care had impacted on service delivery, practitioners' roles, and patients' experiences. The studies reported here were conducted as a realistic evaluation of protocol-based care. Given the lack of published examples, particularly in implementation research, our intention is to describe the application of realistic evaluation, whilst sharing findings of relevance to implementation researchers, managers, and practitioners about standardising care through the use of tools such as protocols, guidelines, and pathways.

### Protocol-based care

As suggested above, the term 'protocol-based care' was developed by policy makers and having emerged relatively recently in policy documents is poorly, but broadly defined as a mechanism for providing clear statements and standards for the delivery of care locally [4]. This definition implicitly conflates protocols, statements, and standards, when arguably these could be conceptually and practically discrete, but it does imply standardisation of care and local delivery. Illot and colleagues suggest that protocol-based care is concerned with staff following 'codified rules'[5]. However, in practice, practitioners are rarely bound to follow guidelines, protocols, and standards, and so 'rules' may not necessarily be a defining feature of protocol-based care *per se*. Because of this lack of clarity, we used protocol-based care as an umbrella term, which encompassed the use of a number of different care processes aimed at standardisation, including protocols, guidelines, care pathways, and algorithms that were being used in service delivery at the time of the study [6,7]. When we embarked on the study, it was unclear whether protocol-based care would be something greater than the sum of its parts [8].

Whilst standardised care approaches such as guidelines and protocols have the potential to mediate the use of research evidence in practice, arguably their effectiveness will be dependent on whether (or not) they are successfully implemented and then routinely used. The challenges of implementing evidence into practice are now well documented in the international literature [9-13]. From a policy perspective, the apparent goal to standardise care assumes a number of things, including that such tools are: are part of the evidence base that practitioners use; are used as intended; and standardisation is an 'ideal' state. Whilst researchers' report efforts to test various implementation strategies within research studies [14,15], we actually know little about how implementation is managed at a local level by those on the ground delivering services on a day-to-day basis.

The other political impetus behind protocol-based care concerned the introduction of the European Working Time Directive [16], which as a statutory regulation has reduced the number of hours that junior doctors work. This, in combination with a shifting policy and service context aimed at flexible service delivery, resulted in health professionals' roles and ways of working evolving, and traditional role boundaries blurring. Politically, protocol-based care was viewed as a mechanism for facilitating the expansion and extension of nurses' and midwives' roles.

Two complementary research studies were conducted in parallel with an overall objective to describe the nature, scope, and impact of protocol-based care in the English NHS, and to determine the nursing, midwifery, and health visiting contribution to its development, implementation, and use, including decision making. As the studies were methodologically complementary, for clarity and consistency with the final report http://www.sdo.nihr.ac.uk/projdetails.php?ref=08-1405-078, throughout the paper we will refer to 'the evaluation' or 'the study.' Additionally, because of the lack of clarity of the term protocol-based care, we use the term 'standardised care approach' to represent the use of a number of different care processes aimed at standardisation.

Whilst becoming an increasingly popular approach to research and evaluation there are few published examples of the use of realistic evaluation in health services research [*e.g.*, [17-20]], and only one that we could find [17] that is directly relevant to the field of implementation research. The following describes our application of realistic evaluation in the study of protocol-based care.

## Methods

### Realistic evaluation

Realistic evaluation has its roots in realism. Realism as a philosophy of science is situated between the extremes of positivism and relativism [21-23] and acknowledges that the world is an open system, with structures and layers that interact to form mechanisms and contexts. Therefore realistic evaluation research is concerned with the identification of underlying causal mechanisms and how they work under what conditions [21-26]. Because causal mechanisms are always embedded within particular contexts and social processes, there is a need to understand the complex relationship between these mechanisms and the effect that context has on their operationalisation and outcome. Pawson and Tilley sum this up as: context (C) + mechanism (M) = outcome (O) [21]. Because these relationships are contextually bound, they are not fixed; that is, particular interventions/programmes/innovations might work differently in different situations and circumstances. So, rather than identifying simple cause and effect relationships, realistic evaluation activity is concerned with finding out about what mechanisms work, in what conditions, why, and to produce which outcomes?

Realistic evaluation was particularly relevant to investigating the practice and impact of protocol-based care. Protocol-based care, a complex intervention in itself, was being studied within the complex system of health care delivery consisting of layers of actors, social processes, and structures. Our research questions called for an understanding of how protocol-based care was being operationalised within the reality of the clinical context, and what sort of impact it might be having on practice, practitioners, organisations, and patients. We were interested in understanding the relationships between the type and nature of particular approaches to protocol-based care (mechanisms of standardisation), within the different clinical settings in which they were being used (context), and what impacts this resulted in (outcomes); *i.e.*, what worked or not. Fundamentally we were interested in finding out the answer to the evaluative question: Protocol-based care: What works, for whom, why, and in what circumstances?

As Tolson and colleagues observe, 'the methodological rules of realistic evaluation are still emerging'. In our experience, Pawson and Tilley provide a set of realistic evaluation principles, rather than methodological rules, or steps to follow. These broad principles include:

1. Stakeholder involvement and engagement.

2. Mechanisms are theories, which are based on a hypothesis or proposition that postulates.... if we deliver a programme in this way or we manage services like this, then we will bring about some improved outcome. Mechanisms are contingent upon contexts.

3. The development and testing of context, mechanism, and outcome (CMO) configurations (*i.e.*, hypotheses/propositions): initial configurations being conjectured CMOs, and refined through the evaluation process (refined CMOs) to generate explanation about what works, for whom, how, and in what circumstances.

4. There is a generative conception of causality -- *i.e.*, not an explanation of the variables that are related to one another, rather how they are associated.

5. Researchers should aim for cumulation rather than replication [21].

Therefore, whilst the operationalisation of realistic evaluation will vary according to the particular evaluation or research study being conducted, the principles outlined above should be evident.

## Findings

### Phase one: theoretical framework, evidence review to propositions

For this study, the process of theory formulation began as a synthesis of policy and research literature; the theories and working propositions (*i.e.*, CMOs) were then refined through data analysis and interpretation. We conducted the evidence review using the principles of realist synthesis [26-28]. Using this approach ensured the study had methodological and theoretical integrity.

**Figure 1:**
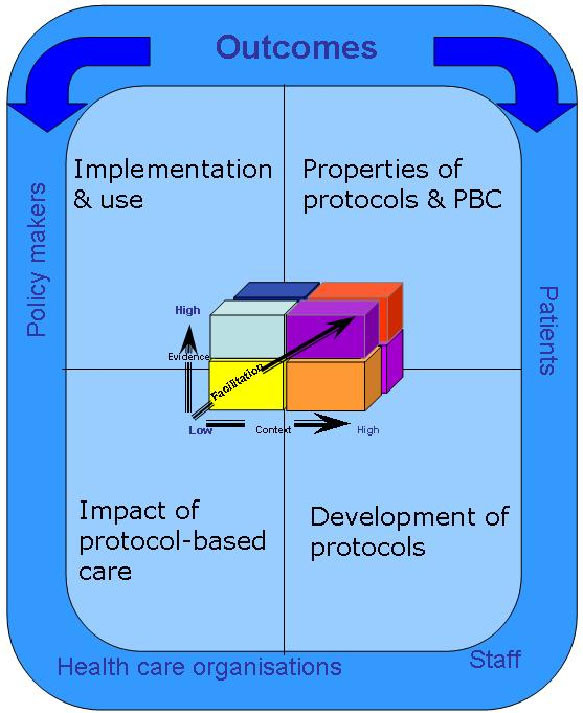
**Theoretical Framework**.

The first stage of the synthesis involved the identification of concepts, programme theories, and subsequent framework development (Figure 1). The construction of the framework was informed by the funder's requirement, an initial review of the literature undertaken for the proposal [6], and key policy developments. The study's theoretical framework integrates various components, including the four areas that play a role in protocol-based care and related impact on stakeholder outcomes: patients, staff, organisations, and policy makers:

1. What are the properties of protocol-based care and protocols?

2. How are protocols developed?

3. What is the impact of protocol-based care?

4. How is protocol-based care implemented and used?

Additionally, implicit in the framework is the notion that protocol-based care is about introducing new practices, which is a function of the nature of the evidence underpinning the new practice (protocol, guideline), the readiness and quality of the context into which they are to be implemented and used, and the processes by which they are implemented. Therefore, the Promoting Action on Research Implementation in Health Service (PARIHS) framework was also embedded into the framework [9,10]. The four theoretical areas needed to be related to outcomes and stakeholder issues; as such each area contained additional review questions:

1. Properties of protocol-based care and protocols:

1a. What is protocol-based care?

1b. What are protocols and what types/models of protocol based care are used in practice?

1c. What patient care issues/topics are covered by protocol-based care?

2. Development of protocols:

2a. How are protocols developed?

2b. What forms of evidence underpin the development of protocols?

2c. How does the method of protocol development affect use?

3. Impact of protocol-based care:

3a. How does protocol-based care impact on patient and organisational outcomes?

3b. How does protocol-based care impact on nurses and midwives?

3c. How does protocol-based care impact on nurses' and midwives' decision-making?

3d. How does protocol-based care impact on multi-disciplinary decision-making and interaction?

4. Implementation and use:

4a. What approaches are used to implement protocols, and how does this impact on their use?

4b. What are the facilitators and barriers to protocol-based care?

These questions were addressed by referring to available literature. Electronic searching including the Cochrane Trial Register, Medline, Embase, Cinahl, Assia, Psychinfo and hand searching was also used. As this literature about standardising care is vast and applying the principle suggested by Pawson [27], searching and retrieval stopped when there was sufficient evidence to answer the questions posed. Literature was reviewed and information extracted using a proforma designed to capture data about the questions in each theory area, and their impact on patients, organisations, and staff.

As part of the review process, propositions were developed to be evaluated in phase two. Propositions were developed by searching for patterns within the literature about a particular theory area related to CMO. For example, in relation to properties of protocol-based care, looking for patterns about what types of properties (mechanisms) of standardised care approaches might impact (outcome) on their use in particular care settings (context)? In practice, because the literature was so variable, it was difficult to trace clear CMO threads, therefore some of the resultant propositions were fairly broad.

By way of illustration the following sections provide a brief summary of the literature within each theory area and linked propositions [29].

### Theory area one: Properties of protocol-based care and protocols

Standardised care approaches are widely used in service delivery and care; however, the term protocol-based care is absent. Similarly, there is little clarity about standardised care approaches, what they are, and a lack of agreement and consistency in the way terms are used. We found that standardised care approaches: localised care delivery through the use of care pathways, protocols, guidelines, algorithms (and other approaches such as patient group directives), and by particularising evidence to the local context; varied in the degree of specificity and prescriptiveness of formalised and/or codified information, and have the potential to involve all members of the health care team, and facilitate the sharing of roles and responsibilities. The following propositions resulted:

1. A clear understanding about the purpose and nature of protocol-based care by potential users will determine the extent to which standard care approaches are routinely used in practice.

2. The properties of standardised care approaches, such as degree of specificity and prescriptiveness, will influence whether and how they are used in practice.

### Theory area two: Development of protocols

Whether standardised care approaches impact on practice and patient care is likely to be partly dependent on the way in which they are developed and the evidence base used in the development process. There is some available guidance on development processes; however this is general, and it is not clear how this has been used to develop standardised care approaches locally. Furthermore, authors who have developed protocols locally tend to provide limited information about development processes. It is therefore unclear how the development process might affect the subsequent use of resulting standardised approaches to care because of limited empirical evidence. The following propositions resulted:

1. Standardised care approaches that are developed through a systematic, inclusive, and transparent process may be more readily used in practice.

2. Standardised care approaches that are based on a clear and robust evidence base are more likely to impact positively on outcomes.

3. Locally developed standardised care approaches may be more acceptable to practitioners and consequently more likely to be used in practice.

### Theory area three: Impact of protocol-based care

The evidence for the impact of standardised care processes on practice, patient and staff outcomes is variable. Even within studies there may be a demonstrable effect on one type of outcome, but no significant changes to others. There are questions about whether it may be the components or characteristics of the particular protocol, or the process of implementation that influence impact, or both. However, there is evidence to indicate that standardised care approaches can be influential, if only to raise awareness about particular issues or as an opportunity to bring clinical teams together [30]. Findings from research also show that protocols can enable nurses' autonomous practice, support junior or inexperienced staff, and can be a vehicle for asserting power [31]. The following propositions resulted:

1. The impact of protocol-based care will be influenced by the type of protocol being used, by who is using it/them, how, and in what circumstances.

2. More senior and experienced clinical staff will be less positive than junior and/or inexperienced nurses about using standardised care approaches.

3. The impact on decision making will be influenced by practitioners' perceived utility of standardised approaches to care.

4. Protocol-based care will impact on the scope and enactment of traditional nursing roles. Protocol-based care has the potential to enhance nurses' autonomy and decision-making latitude.

5. The impact on patient care will be influenced by the characteristics and components of the protocol and factors in the context of practice.

### Theory area four: Implementation and use

Approaches to implementation, including clear project leadership, that have the scope to identify and address the complexities of use may be more successful in encouraging uptake than those that do not. Furthermore, integrating standardised care approaches within existing systems and processes may facilitate their use. In addition, certain contextual factors may facilitate or inhibit the use of standardised care approaches, although what these factors are requires further investigation. The following propositions resulted:

1. Interactive and participatory approaches and strategies to implement standardised approaches to care may influence whether or not they are used in practice.

2. The support of a project lead may increase the likelihood of the ongoing use of standardised care approaches.

3. Embedding the standardised care approach into systems and process may facilitate use, but there is a lack of evidence about how this might work for different groups and in different contexts.

4. Some contexts will be more conducive to using standardised care approaches than others, but it is unclear what might work in what circumstances and how.

### Phase two: Testing propositions through case studies

Case study [32,33] was used because it is methodologically complementary to realistic evaluation, which advocates the use of multiple methods to data collection, and recognises the importance of context. As with case study, realistic evaluation calls for making sense of various data sets (*i.e.*, plurality) to develop coherent and plausible accounts. The refinement of the propositions required descriptive and explanatory case study. Additionally, in order to assist in explanation building and transferability of findings, multiple comparative case studies were included.

A 'case' was defined as a particular clinical setting/context, for example, a cardiac surgical unit (CSU), and the 'embedded unit' of that case the use of a particular standardised care approach, for example, the care pathway. Sites were purposively sampled in order to maximise rigour in relation to applicability and theoretical transferability [34]. Criteria for selection included reported active engagement in protocol-based care activity, a requirement to study the use of a variety of standardised care approaches, and to study this use in different clinical settings in depth over time. Sites selected within England are listed in Table 1.

**Table 1:** Clinical sites selected for study.

Site	Description
CSU	Cardiac Surgical Unit
WIC	Walk-in Centre
PAC	Pre-operative Assessment Clinics
BC	Birth Centre
GPS	General Practice Surgery
CMU	Cardiac Medical Unit
DC	Diabetic and Endocrine Clinic

Pawson and Tilley [21] argue that realistic evaluators should not be pluralists for pluralism's sake, but that methods should be chosen to test the hypotheses/propositions. Given the broad scope of the initial propositions and a desire to capture how standardised care approaches worked *in situ*, we used a combination of methods, including those from ethnography:

1. Non-participant and participant observation of nursing and multi-disciplinary activities related to the use of standardised care approaches. Observations and discussions were recorded in field notes and/or audio-recorded as appropriate.

2. Post-observation interviews guided by issues arising from observations.

3. Key stakeholder interviews exploring views in general about the use, influences on use, and impact of standardised care approaches. Interviews were audio-recorded and later transcribed in full.

4. Interviews with patients about their experiences of standardised care.

5. Tracking of patient journeys in which patients were interviewed a number of times during their contact with the service.

6. Review of relevant documentation, such as copies of guidelines, protocols, and pathways.

7. Field notes written during and after each site visit.

Data were collected in sites for between 20 and 50 days. Study participants and data collected are presented in Tables 2 and 3.

**Table 2:** Study participants.

Participant type/site	CSU	WIC	PAC	BC	GPS	CMU	DC	Total
**Clinical nursing staff**	13	11	14	7	7	20	20	92
**Health visitors**	0	0	0	0	3	0	0	3
**Midwives**	0	0	0	0	3	0	0	3
**Medical staff**	3	3	7	2	4	4	2	25
**Managers**	2	1	1	2	3	0	0	9
**Non-clinical staff**	5	1	1	1	3	0	1	12
**Administrative staff**	1	1	0	0	1	0	0	3
**Patients**	8	7	6	6	10	4	13	54
**Allied healthcare professions**	2	1	1	0	0	0	0	4
**Total**	**34**	**25**	**30**	**18**	**34**	**28**	**36**	**205**

**Table 3:** Data collected within and across sites.

Type of data collection	CSU	WIC	PAC	BC	GPS	CMU	DC	TOTAL
**Non-participant observations**	11	8	10	4	11	21	20	85
**Post-observation interviews with healthcare professional**	10	7	8	4	9	21	20	79
**Post-observation interviews with patients**	8	7	8	6	10	4	13	56
**Follow-up interviews with patients**	4	2	2	0	2	0	0	10
**Interviews with key staff**	15	8	14	14	15	0	0	66
**Review of relevant documentation**	yes	yes	yes	yes	yes	yes	yes	yes
**Field notes (on days present)**	21	22	17	32	16	50	50	208

### Ethics

Multi-site Research Ethics Committee (MREC) approval was sought and given. Each potential participant was given information about the study and an appropriate period of time allowed to lapse to before written consent was sought. Anonymity was assured by each site and all participants were given an identity code.

### Approach to analysis

As this evaluation was a 'snap shot' of the use of standardised care approaches within sites, we used the analysis stage to test and refine propositions between site visits, and then in the final stages across data sets and sites, *i.e.*, we did not capture any changes within sites over time.

Using a process of pattern matching and explanation building for each CMO, evidence threads were developed from analysing and then integrating the various data. The fine tuning of CMOs was a process that ranged from abstraction to specification, including the following iterations:

1. Developing the theoretical propositions at the highest level of abstraction -- what might work, in what contexts, how, and with what outcomes, and are described in broad/general terms above. For example, 'embedding the standardised care approach into systems and process (M1) may facilitate use' (O1) at least in some instances (C1, C2, C3...).

2. Data analysis and integration facilitated CMO specification ('testing'). That is, we refined our understanding of the interactions between M1, O1, C1, C2, C3. For example, data analysis showed that in fact there appeared to be particular approaches to embedding standardised care approaches (computerisation) (now represented by M2), that had an impact on their routine use in practice (now represented by O2), in settings where nurses were autonomous practitioners (an additional C, now represented by C4). These new CMO configurations (*i.e.*, propositions) were then 'tested' with data from other sites to seek disconfirming or contradictory evidence.

3. Cross-case comparisons determined how/whether the same mechanisms played out in different contexts to produce different outcomes.

This process resulted in a set of theoretically generalisable features addressing our overarching evaluation question: Protocol-based care: what works, for whom, why, and in what circumstances? The following sections describe some of the findings that emerged from the analysis.

### The nature of protocol-based care

**Figure 2:**
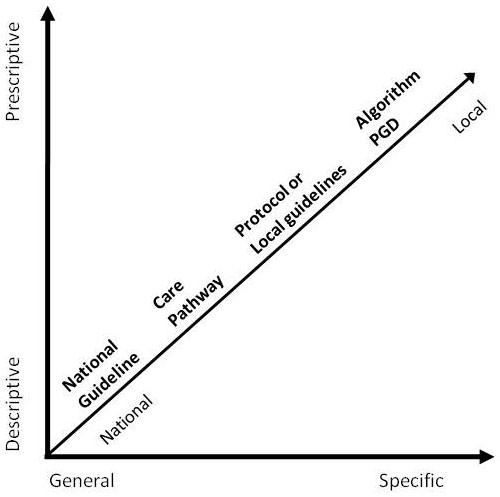
**Conceptualisation of frequently used standardised care approaches**.

Protocol-based care encompassed a variety of different standardised care approaches, patient conditions, and care delivery often within single sites; however, it was not a term that participants recognised. Data shows that protocol-based care was no greater than delivering (some) care with the use of particular standardised care approaches. In the reality of practice, the use of standardised care approaches was patchy, and influenced by individual, professional, and contextual factors. The most commonly used approaches were care pathways, local guidelines, protocols, algorithms, and patient group directives (PGD; medication prescribing protocol). Each of these was perceived, and did in practice, have differing levels of prescriptiveness, specificity, and applicability. These approaches and their characteristics have been plotted in Figure 2.

Data shows that protocol-based care appeared not to be greater than the sum of its parts [8]. The initial propositions (conjectured CMOs) that were developed from the evidence review only partially corresponded to the findings that emerged during analysis. From the iterative analysis process of scrutinising mechanisms, context, and outcomes (*i.e.*, propositions), we were able to draw out what works, for whom, how, and what circumstances in relation to the use of standardised care approaches (refined CMOs). This is summarised in Table 4 and elaborated on in the text below by integrating data to provide some illustrative examples of what worked, for whom, how, and in what circumstances (see full report for a comprehensive account of the findings with data excerpts [29]).

**Table 4:** What works, for whom, how, and in what circumstances.

**What works**	**New ways of working**: standardised care approaches that supported the development of new services such as nurse and/or midwife led care were consistently used.
	**New roles**: standardised care approaches that enabled the extension of nursing roles tended to be used.
	**Location and visibility**: standardised care approaches that are readily available and are highly visible are more likely to be used.
	**Incentives**: standardised care approaches linked to financial rewards were consistently used.
	**Buy-in**: generally when the whole team (multi/uni-disciplinary) has been actively involved in the development of a standardised care approach it tends to be used.
	**Making a difference**: standardised care approaches that practitioners perceived as making difference to their practice and patients were used.
**For whom**	**Mainly nurses, midwives, and health visitors**: despite existence of multi-disciplinary standardised care approaches, medical staff rarely used them (for exceptions see below).
	**Medical staff**: some junior doctors found standardised care approaches useful. General Practitioners consistently used Quality Outcomes Framework related protocols.
	**Students, newly qualified, temporary, and new staff**: standardised care approaches were perceived to be a useful heuristics to organising care for those who do not have experience (usually nurses but also medics and Allied Health Professionals).
	**Nurses taking on new roles**: standardised care approaches gave nurses confidence for delivering care autonomously (*e.g.*, nurse/midwife-led clinics and services).
**How**	**Explicit use**: some standardised care approaches were being used on-screen and shared with the patient -- usually as checklists or prompts. Additionally they could be useful sources of information for some staff.
	**Implicit use**: some standardised care approaches were not explicitly referred to, but their principles may guide care.
	**Embedded in documentation**: some standardised care approaches were embedded in routine documentation, sometimes replacing or complementing patient's notes.
	**Embedded in IT systems**: some standardised care approaches were part of routine systems and worked effectively as a prompt.
**In what circumstances**	**Nurse/midwife**-**led services**: standardised care approaches supporting the running of nurse and midwife-led services and clinics were more likely to be used.
	**Protection from litigation**: when nurses were practising outside their traditional scope of practice standardised care approaches were consistently used because they provided a safety net.
	**Mandatory**: when the use of standardised care approaches was compulsory they were consistently used, and supported with regular audits and training.
	**Financial reward**: for outcomes of use, encouraged commitment to and use of linked protocols.
	**Ongoing project lead**: the existence of such a role seemed to facilitate active involvement of the multi-disciplinary team. The lead also enabled on-going monitoring of use.
	**Strategic support**: for the development and sustained implementation of standardised care approaches.

### Example one: What works, for whom, how, in what circumstances -- extending roles and autonomy

There was clear evidence to show that standardised care approaches enabled the extension of traditional roles, and facilitate autonomous practice, which in turn resulted in more nurse and midwifery led care and services. These were perceived to be positive developments by doctors, nurses, and midwives. This finding came from data collected in the walk-in-centre (WIC), pre-assessment clinics (PAC), birth centre (BC), GP surgery (GPS), and diabetes clinic (DC), in the following ways:

WIC -- The clinical guidelines and algorithms facilitated the development of nurses' skills in examining and diagnosing. The patient group directives enabled them to extend their role to treating patients without the need to consult GP colleagues to obtain prescriptions.

PAC -- The pre-operative assessment guidelines and protocols supported nurse-led clinics enabling them to make decisions about what tests to order, how to interpret results, and ultimately to make decisions about fitness for surgery.

BC -- The normal labour pathway supported the development of a midwifery-led service for healthy pregnant women.

GPS -- Protocols enabled nurses to independently run clinics on the management of chronic diseases such as asthma, diabetes, and hypertension. Nurses were responsible for diagnosing, monitoring patient status, and recommending appropriate medications.

DC -- Protocols facilitated clinical nurse specialists to run clinics and performing tests and procedures independently.

It is difficult to determine whether it was the standardised care approaches that facilitated autonomous practice or the practice environment that supported nurses' practising autonomously. In this study, nurses were able to practice autonomously because of their role (they tended to be more senior, and/or be independent practitioners, *e.g.*, clinical nurse specialists, midwives and health visitors) and because services were nurse-led. The development and introduction of standardised care approaches facilitated the enactment of both nurse-led service delivery and to work outside their traditional scope of practice. Findings showed that where nurses practised autonomously they were able to deliver more streamlined care because on a patient-by-patient basis they did not have to refer to, or follow up with doctors. A perhaps unintended consequence was the perceived protection value available standardised care approaches offered if nurses' judgements were questioned; they were considered to be a 'safety net.' In contrast, some doctors interviewed felt they provided a 'false sense of security.'

### Example two: What works, for whom, how, in what circumstances -- use and visibility

**Figure 3:**
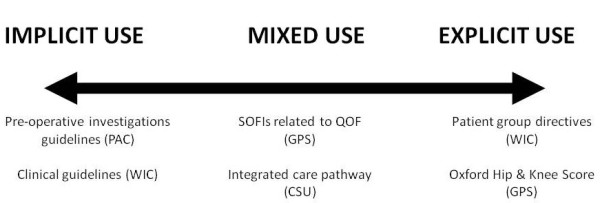
**Examples of how standardised care approaches were used**.

Observing practice was useful in determining how and if standardised care approaches were being used in the practice settings. Overall, the use of standardised care approaches across all sites could be described on a continuum ranging from implicit to explicit use (see Figure 3). For example, there were instances where during their interactions with patients, nurses, and doctors explicitly referred to protocols (*e.g.*, as a checklist or reference). In contrast, there were many occasions where it was not obvious that available standardised care approaches were being used to explicitly guide care. For example, in the PAC clinics whilst there were protocols for ordering patient tests, nurses did not always refer to them, but used principles from them to apply to particular patients, justifying why they had not used the protocol in those instances.

The location of the standardised care approach and its level of visibility influenced how and whether it was used. In settings where they were more visible, physically close to the patient-practitioner interaction, and/or easily accessible, they tended to be referred to more often. For example, algorithms in the walk in centre were computer-based and were often used as an onscreen-prompting tool during interactions with patients. A similar finding emerged from GP site data where most staff routinely used the onscreen protocols (SOFIs) related to the Quality and Outcomes Framework (QOF). In the walk-in centre some nurses had copies of PGDs that fitted into their pockets or bags so that they could be quickly and easily referred to at the point of care. Furthermore, embedding the care pathways in documentation in both the cardiac surgical unit and the birth centre ensured that they were used routinely by the relevant professionals. In sites where these mechanisms were not in place, the explicit use of the standardised care approaches was patchy. For example, in the cardiac-thoracic unit, nurses described the location of guidelines, policies, and protocols as scattered in various areas, and mainly hidden from view. Similarly, in the pre-operative assessment clinics where the guidelines and protocols were in a paper-based manual, they were rarely referred to.

### Example three: What works, for whom, how, in what circumstances -- making a difference

Where practitioners could see that the use of the standardised care approaches were making a difference to their practice, patient care, or service delivery, they tended to be more consistently used. In the GP site, opinion was unanimous that the use of the QOF-related protocols had improved the standard of patients' care; this perception was supported by the consistent achievement of targets and high QOF points, which provided a financial incentive to continue use.

In other sites, the ability of nurses to be able to practise autonomously and in extended roles appeared to provide a motivation to continue to use available protocols and guidelines. This was particularly the case in the walk-in centre with the use of the PGDs and algorithms, in the birth centre where care was completely midwifery led, and in the GP practice where nurses, midwives, and health visitors were running clinics.

### Example four: What works, for whom, how, in what circumstances -- prescriptiveness versus flexiblity

The flexibility of the standardised care approaches appeared to impact on the way that they were used; however there are contradictory findings with respect to flexibility. For example, interviewees in the cardiac surgical unit felt that the care pathway was inflexible because it could not be used with patients who were complex cases (the care pathway had been developed for 'straightforward' cases). In contrast, nurses in the walk-in centre were using algorithms, which they described as prescriptive (and so not flexible) and apart from a small number of nurses, they were consistently used, even if only as a checklist at the end of a procedure or patient interaction. Similarly, protocols related to QOF, whilst prescriptive, were used by most staff in the practice. Whether it was the flexibility of the standardised care approach *per se *that influenced the type and amount of its use, or factors such as the motivation for using them --for example, incentives and being able to run a nurse-led service independently -- is difficult to unravel. However, this finding highlights that context of use is important, what might work in one setting may work differently in another.

### Example five: What works, for whom, how and in what circumstances -- information sources

For new and/or junior doctors, nurses, and midwives, standardised care approaches of all types were perceived to be useful information resources. In contexts in which there were frequent staff changes, and/or reliance on agency practitioners, local standardised care approaches provided information about what was expected in terms of care delivery and standards in that particular setting. As a result, in some sites they were included in induction materials and formed part of competency assessments. In contrast, there was an expectation that more senior staff, by virtue of their experience, should already know that information contained in such tools. Nurses and midwives in this study, particularly those with more experience, either did not refer to them, or used them flexibly. They tended to privilege their own experience, or the experience of others, instead of referring to available standardised care approaches. Nurses, if unsure, tended to refer to human sources of information (rather than available standardised care approaches), such as a credible and knowledgeable colleague.

### Example six: What works, for whom, how and in what circumstances -- team functioning

Findings show that standardised care approaches had no obvious effect on team functioning. In fact, there is evidence to suggest that standardised care approaches formalised respective roles, rather than enhanced teamwork. For example, within the cardiac surgical unit, the integrated care pathway, whilst it had been designed to become a permanent part of the multi-disciplinary record of care, had been colour coded so that each professional's section was easily identifiable. This resulted in the different professionals rarely consulting sections that were not their own; a practise seen during observations. An alternative view is that this approach clarified the contribution that each team member made to the patient's journey through cardiac surgery (even if it did not appear to enhance team working), and indeed the development of health visiting guidelines within the GP surgery had been viewed as an opportunity to clarify roles and responsibilities around skills.

In other sites with the exception of the GP surgery and the use of QOF-related protocols, and some junior doctors, generally medics were not using available standardised care approaches even if they were applicable to them. The common perception amongst both doctors and nurses/midwives was that the use of standardised care approaches was a nursing and midwifery initiative.

## Discussion

Given the goal of realistic evaluation, *i.e.*, to uncover what works, for whom, how, and in what circumstances, its application to this research was a good fit. We were funded to find out whether protocol-based care had impacted in service delivery, in what ways, for whom, and how. Additionally, how different service delivery contexts might affect the use of different types of standardised care approaches was an important consideration. In recent years, there has been a growing interest in the study of context within implementation research [35-39]. Therefore, methodological approaches that focus attention on the study of context are timely. Within realistic evaluation, the fundamental proposition is that the effect of a mechanism (*e.g.*, particular standardised care approach's mechanism of action) is contingent upon context (*e.g.*, particular type of service delivery, nurse role etc.); that is, the outcome is a product of both mechanism and context. So a realistic evaluator's job is concerned within finding out about what the contingencies between mechanism and contexts are. For example, in this study, we found that algorithms and patient group directives (mechanisms) being used within nurse-led service delivery (context) resulted in a more streamlined patient journey (outcome). However, that is not to say that the same finding would result in different care delivery settings; this would need to be tested through a process of cumulation [21] (discussed below), which we did not have the resources to accomplish in this study.

A further strength of realistic evaluation is in the potential for developing explanatory theory. As previously observed, there has been a lack of attention to theory in implementation and knowledge translation research [40-42], furthermore, theory use and development to date has been mainly positivistic (and isolated from context), with fewer examples using constructivist or interpretive approaches. As Pawson and Tilley [21] state, 'realism has a unique way of understanding the constituents of theory,' not in an x causes y sort of way, but in a way that is described as generative causation between mechanism, context, and outcome [24]. Thus, one engages with theory at the start of the evaluation process through the development of conjectured CMO threads; they are the theories of change that one tests and refines throughout the evaluation process. The potential therefore with using realistic evaluation within implementation research includes the interpretive development of middle range theory about, for example, why some approaches/interventions work.

Given the lack of published examples of the use of realistic evaluation in healthcare research (particularly at the start of this project), and a book whilst innovative, is not a methodological recipe for doing realistic evaluation [21], we found that the greatest challenge with using this approach was in its operationalisation. The principles or 'rules' as they are referred to within the realistic evaluation text are helpful but they do not tell you how to undertake evaluation research. In fact, Pawson and Tilley are clear that they are sensitive to the idea of laying down the rules of realistic evaluation inquiry, but stress that it is only by trying them out in practice that methodological progress will be made. So, whilst this affords the researcher some latitude, at times it can feel like being part of a natural experiment, moving between principle and practice. As more examples are published and particularly those that are explicit about how the approach was operationalised, it is likely that, as Pawson and Tilley aspire to, the 'methodological rules of realistic evaluation will become the medium and outcome of research practice.'

A particular challenge in this study was in being able to clearly define mechanisms, and distinguish between what was a mechanism and what was context. For example, was the consistent use of the electronic protocols related to the QOF by general practice surgery staff a mechanism (for monitoring patient wellbeing), or was the fact that the consistent use of these protocols was determined by the fact that use resulted in financial reward? In this example, it was not clear whether the incentive is context, or the underlying mechanism of use. Theoretically, a mechanism is the answer to the question 'what is it about a program' that makes it work; this could be observable or hidden, and at micro and/or macro levels; so on that basis with this example the incentive could be both a mechanism and context and is dependent upon the level of abstraction. Byng and colleagues [18] had similar challenges that they resolved by returning to the philosophical basis of realism, which focuses attention on the idea that there may be more than one mechanism in operation at the same time. As such, what is important is the process of developing, testing, and refining the CMO configurations because it this procedure that has the potential to unearth the various permutations, which helps us to better understand what is, or has, occurred. Within this study, our resources meant that the testing of the conjectured CMO configurations ended after only one examination. Ideally, we should have continued to test and refine the configurations over more than one cycle of data collection. Indeed if this had been possible, we may have been able to resolve some of the challenges we had between identifying mechanisms and contexts.

In further critiquing our use of realistic evaluation, other operational and methodological issues arise. First, due to funding constraints we were unable to carry out a full realist synthesis [27], instead the principles were applied. This could have resulted in less specific, more general propositions than if we had the opportunity to develop a more comprehensive, possibly more in-depth synthesis. We were also limited by the quality of the existing evidence base, with many papers lacking essential detail about the use, development, and impact of standardised care approaches. Subsequently, testing and refining these propositions in phase two may have resulted in findings with fewer nuances. We hope to have counteracted this by drawing on and integrating various data sources that resulted in a rich picture. Operationally, iteratively juggling the various data sources to move from propositions to a summary of what works, for whom, how, and in what circumstances required flexibility, and a continual process of checking and discussion. It is possible, given the interpretative nature of this approach, that other teams might arrive at different conclusions. Our audit trail is clearly documented [29], and therefore could be followed by others; at face value, and from our knowledge of the field, we are confident our conclusions are sound.

Pawson and Tilley's argument is that replication is an inappropriate concept for evaluating complex interventions and processes. Given that realistic evaluation is concerned with uncovering the contingencies of mechanisms and contexts, exact replications are unlikely to be achievable. Instead, the idea of cumulation [21] is offered as a way of building insight or ideas across and between cases for theory development rather than empirical generalisation. Despite only one cycle of data collection within each site, we have started to build some explanatory theory from considering data across sites, which represents the use of standardised care approaches as a function of: individual practitioner attitudes and level of clinical experience; the degree of support their use offers roles and/or practice, and/or service delivery; the degree of visibility and embeddedness of the standardised care approach(s) within the system/organisation; how active implementation processes/activities are; and the availability of internal or external reward for ongoing use.

This theory now needs further refinement and 'testing' across different types of sites and data from other studies.

## Summary

This paper provides an overview of the application of realistic evaluation in attempting to uncover how various types of standardised care approaches are being used in the reality of clinical settings. Whilst sometimes challenging to operationalise, the approach provided a useful framework with which to make sense of the multiple factors that were simultaneously at play and being observed through various data sources. Two practical lessons we have learnt through applying this approach include the need to ensure the project management plan includes ample time for discussion and debate, and developing flexible, yet transparent approaches for tracing iterative processes. Methodologically, we have also learned lessons. Because realistic evaluation is an interpretive approach, it is important to be clear, from the outset how one is defining CMOs. Our later challenges with delineating mechanisms and contexts within primary data may have been helped if we had had more discussion about definitions earlier on. The idea of CMO makes intuitive sense to implementation science, as does the notion that one cannot separate out outcome from mechanism of action and operationalisation within particular contexts that are in a constant state of flux. However, this view, and therefore perhaps this approach, will likely appeal to those who have more leaning towards interpretive, rather than deductive approaches.

For this study, realistic evaluation provided an extremely useful framework for helping us develop explanations and present them in a coherent way; as Pawson and Tilley [21] suggest, these now need to be marshalled into a 'wider cycle of enlightenment' about the use and impact of standardised care approaches in service delivery and patient care.

## Competing interests

The authors declare that they have no competing interests.

## Authors' contributions

JRM conceived, designed, secured funding, was involved in and supervised all aspects of the research and led the drafting and revision of the manuscript. MF coordinated and took a lead role in data collection and analysis, and commented on drafts of the paper. DB contributed to the design of the study, led data collection and analysis in one site, participated in the analysis processes for the project as a whole, and commented on drafts of the paper. KS contributed to the design of the decision making study and commented on drafts of this paper. All authors approved the final manuscript.
